# Regulation and Function of RFRP-3 (GnIH) Neurons during Postnatal Development

**DOI:** 10.3389/fendo.2015.00150

**Published:** 2015-09-24

**Authors:** Matthew C. Poling, Alexander S. Kauffman

**Affiliations:** ^1^Department of Reproductive Medicine, University of California, San Diego, La Jolla, CA, USA

**Keywords:** RFRP-3, GnIH, development, Gpr147, Rfrp, reproduction, puberty

## Abstract

RFamide-related peptide-3 (RFRP-3) [mammalian ortholog to gonadotropin-inhibiting hormone (GnIH)] potently inhibits gonadotropin secretion in mammals. Studies of RFRP-3 immunoreactivity and *Rfrp* expression (the gene encoding RFRP-3) in mammalian brains have uncovered several possible pathways regulating RFRP-3 neurons, shedding light on their potential role in reproduction and other processes, and pharmacological studies have probed the target sites of RFRP-3 action. Despite this, there is currently no major consensus on RFRP-3’s specific endogenous role(s) in reproductive physiology. Here, we discuss the latest evidence relating to RFRP-3 neuron regulation and function during development and sexual maturation, focusing on rodents. We highlight significant changes in RFRP-3 and *Rfrp* expression, as well as RFRP-3 neuronal activation, during key stages of postnatal and pubertal development and also discuss recent evidence testing the requisite role of RFRP-3 receptors for normal pubertal timing and developmental LH secretion. Interestingly, some findings suggest that endogenous RFRP-3 signaling may not be necessary for the puberty timing, at least in some species, forcing new hypotheses to be generated regarding this peptide’s functional significance to sexual maturation and development.

## Introduction

Since their identification 15 years ago, both gonadotropin-inhibiting hormone (GnIH) and its mammalian ortholog, RFamide-related peptide (RFRP-3), have become important research focuses of neuroendocrinologists and reproductive biologists. When given exogenously, both peptides potently inhibit the hypothalamic–pituitary–gonadal axis, and numerous studies have studied the *in vivo* and *in vitro* effects of GnIH and RFRP-3 on luteinizing hormone (LH) secretion (1–6). In rodents, as with most mammals, the neurons that produce RFRP-3 are a scattered population localized exclusively within and immediately adjacent to the hypothalamic dorsal-medial nucleus (DMN). This was first characterized by *in situ* hybridization (ISH) for *Rfrp* mRNA (7) and confirmed by immunohistochemistry with RFRP-3 or GnIH antibodies (2, 8).

In rodents, the primary focus of this review, RFRP-3 is thought to regulate LH secretion through inhibition of gonadotropin-releasing hormone (GnRH) neurons rather than by direct action on the pituitary. This model is supported by data, collected primarily in adult animals, showing that RFRP-3 neural fibers appose GnRH neurons (2, 9, 10), RFRP-3’s high affinity receptor, Gpr147, is expressed in some GnRH neurons (11), GnRH neuron electrical firing changes when RFRP-3 is applied to hypothalamic explants (4, 5), and RFRP-3 treatment suppresses LH secretion in a GnRH-dependent manner (10). RFRP-3 may also regulate LH secretion through additional indirect circuits in the brain, as only a subset of GnRH neurons in rodents actually express *Gpr147* mRNA (11). For example, RFRP-3 signaling may modulate the activity of arcuate kisspeptin neurons, which also express the Gpr147 receptor (12), or other upstream neuronal populations. Interestingly, there are two reports of RFRP-3 stimulating LH secretion in hamster species (13, 14), suggesting that in seasonal rodents, RFRP-3 may have stimulatory and inhibitory roles in reproduction. Similar stimulatory effects of RFRP-3 have not been reported in mice or rats in adulthood or development.

## How do RFRP-3 Immunoreactivity and *Rfrp* mRNA Levels Change in the Brain during Development?

RFamide-related peptide immunoreactivity and *Rfrp* mRNA are first detectable in the rat hypothalamus on embryonic day 16 (E16) or E17 (15). Using BrdU labeling to mark neurogenesis, we know that RFRP-3 immunoreactive neurons are born as early as E12, with most RFRP-3 neurons born on E13-E14 (16), consistent with the neurogenesis of the DMN region itself (17). There are minimal detectable RFRP-3 projections during the embryonic stage, as RFRP-3 fibers are nearly absent before or at birth (15). However, by the second and third postnatal weeks, RFRP-3 immunoreactive fibers are clearly visible in some proximal hypothalamic nuclei, such as the arcuate, lateral hypothalamic area, and paraventricular nuclei, as well as non-hypothalamic sites, such as the thalamus and midbrain (15). RFRP-3 fibers do not reach some of their more distal targets, such as the spinal cord, until after puberty is complete (15).

The first two studies to examine postnatal developmental changes in neural *Rfrp* mRNA in rodents used qPCR on either hypothalamic dissections or micropunches. Substantial increases in *Rfrp* mRNA between neonatal/juvenile and peripubertal rats were observed in both sexes (18, 19). However, Iwasa’s study showed a significant decrease in *Rfrp* expression after puberty in male, but not female, rats, whereas Quennell and colleagues reported no significant decrease for either sex between peripubertal ages and adults.

**Figure 1:**
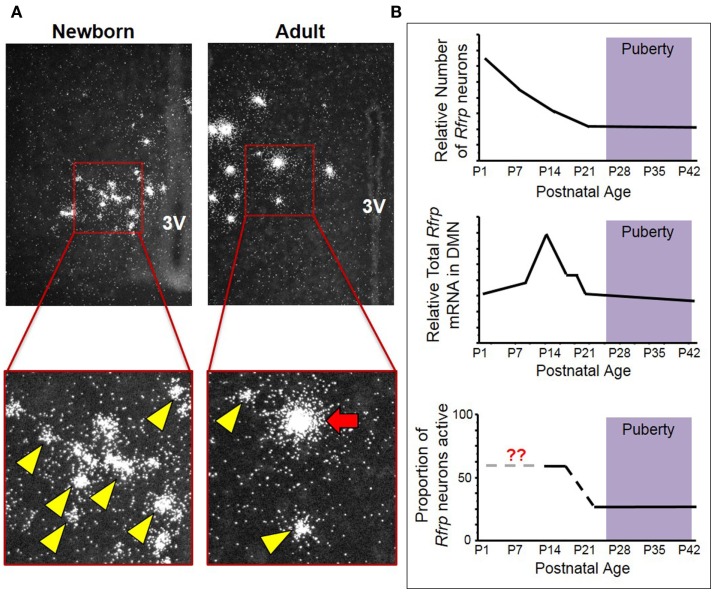
**Changes in neural *Rfrp* expression during postnatal development in mice**. **(A)** Between the day of birth and adulthood, there are significant changes in the expression of certain subtypes of *Rfrp* neurons (categorized based on their *Rfrp* expression levels). Low-expressing (LE) *Rfrp* cells (yellow arrowheads) are the predominate subtype at birth whereas high-expressing (HE) *Rfrp* cells (red arrows) are first detectable during the 2nd week of postnatal life and remain present through adulthood. **(B)** Three graphic representations of *Rfrp* cell number, total *Rfrp* expression levels in the brain, and *Rfrp* neuronal activation (measured by c-Fos co-expression) during rodent postnatal development. P = postnatal day. The purple shading represents the approximate ages when puberty occurs.

Our lab also examined *Rfrp* expression over development by measuring *Rfrp* levels in newborn and adult mice by ISH. We found that not only did *Rfrp* cell number change markedly over postnatal development, but the levels of *Rfrp* mRNA in each cell was dramatically different between the two ages. At birth, there were numerous *Rfrp* neurons detected, but in adulthood, the number of detectable neurons expressing *Rfrp* was notably less (Figure 1). However, the relative amount of *Rfrp* mRNA per neuron, indicated by the number of silver grains per individual cell cluster, was higher in adults. This dramatic change in *Rfrp* mRNA levels per cell demonstrated that while *Rfrp* cell number is *decreasing* during postnatal development, each *Rfrp* cell is a producing, on average, *more Rfrp* mRNA as the animal ages.

To further investigate this intriguing developmental change, ISH was performed for *Rfrp* mRNA at multiple postnatal ages in mice, and each individual *Rfrp* cell was categorized as either low expressing (LE) or high expressing (HE) based on the level of mRNA being expressed by that cell (11, 20). We found that the total number of detectable *Rfrp* cells was highest at birth and significantly dropped through all postnatal ages, with the fewest *Rfrp* neurons present in adulthood. This developmental pattern of *Rfrp* neurons was the same for both sexes (20). Interestingly, the total amount of *Rfrp* mRNA present in the DMN increased around PND 6 and peaked at PND 12 before dropping again at subsequent older ages. This robust juvenile increase in total *Rfrp* mRNA reflects a large developmental increase in the number of identifiable HE *Rfrp* cells, which substantially increase in prevalence and peak around PND 12 before dropping significantly in number at older pubertal and adult ages (20). Conversely, the number of LE *Rfrp* cells decreases slowly and steadily from birth to adulthood, being lowest in adulthood (20). Currently, the phenotypic and functional difference between HE and LE *Rfrp* cells is unknown. However, the HE cell population appears to be the more responsive of the two subtypes to physiological challenges, such as sex steroid treatments and leptin deficiency, as HE cells change more severely than the LE subpopulation (11, 20).

We also examined later postnatal ages in female mice around puberty, using a detailed day-to-day time course. The total number of *Rfrp* cells was found to decrease around PND20-21, around weaning and ~1 week before the onset of female puberty (21). However, this outcome differs from the previously published qRT-PCR rat experiments (18, 19), as neither of those studies showed a consistent decrease in *Rfrp* expression between prepubertal and adult animals. Additionally, the observed juvenile increase in total *Rfrp* expression in mice appears to occur later in development in rats, even when accounting for different developmental timelines between rats and mice. In mice, using ISH, *Rfrp* expression peaks in the second week of life and drops to adult-like levels by the end of third week of postnatal life, about a week before external markers of puberty are noticeable. In rats, *Rfrp* expression increases steadily during postnatal and pubertal development, and only drops to adult-like levels at ages after puberty is completed [note: a significant post-pubertal decrease was only observed in male rats (19)]. However, a newer experiment using qRT-PCR to measure *Rfrp* expression in brains of female mice produced a similar pattern as in our findings: total *Rfrp* expression increases in prepubertal mice then drops at the time of vaginal opening (22). We note that all values in that study were normalized to *Rfrp* expression on PND 24, which is after the age when most *Rfrp* gene expression changes are typically observed in mice in our ISH experiments. Furthermore, those data are from animals that were pooled based on the day they completed vaginal opening, rather than a specific postnatal day. While the implications of the discrepancies in these developmental profiles are currently unclear, the consistent finding is that there is a marked upregulation of RFRP-3 between birth and puberty in rodents. This conclusion is supported by both mRNA and protein data in several species, but the functional significance of this developmental change remains to be determined.

## What Regulates Developmental Changes in Neural *Rfrp* Expression?

Few studies have moved beyond routine descriptive characterization of changes in RFRP-3 or *Rfrp* expression during postnatal life. One study questioned whether the majority of changes observed in *Rfrp* expression and cell number during postnatal development reflected neuronal apoptosis as a possible underlying mechanism (11). Using knockout mice deficient in BCL2-associated X protein (BAX, a major factor causing neuronal apoptosis), we found, in adult male mice, a small but significant increase in the total number of *Rfrp* cells present in the brain compared to WT mice. This higher number of overall *Rfrp* cells was primarily due to increased numbers of detectable LE *Rfrp* cells. However, while the adult BAX KO mice had more total *Rfrp* cells than WTs, the observed difference in cell number was not nearly as great as the difference seen between the day of birth and adulthood in normal WT mice. Thus, because adult BAX KO males did not have a newborn-like number of *Rfrp* neurons, it appears that BAX-mediated apoptosis is not solely responsible for the large overall decrease in cell number seen during development. Therefore, in addition to a minor effect of apoptosis via BAX, other regulatory factors or developmental processes must also be involved in modifying the temporal expression of *Rfrp* neurons throughout development.

Since neuronal apoptosis (via BAX) cannot completely explain the decrease in *Rfrp* neuron number during postnatal development, these neurons are likely undergoing regulatory changes in *Rfrp* expression that (1) decrease the *Rfrp* mRNA expression in some cells to a degree that makes them undetectable by ISH while (2) simultaneously increasing *Rfrp* expression in a subset of neurons, thereby generating the HE *Rfrp* cell population that is virtually absent at birth but which emerges in juvenile life. The mechanisms causing this maturation of the *Rfrp* system may be intrinsic to this neuropeptide cell population, but more likely, an extrinsic factor is acting on the *Rfrp* neuron population to dictate specific changes in gene expression in the various subtypes of *Rfrp* neurons.

A factor that was hypothesized to regulate this developmental change was the adipocyte hormone, leptin. Leptin is well known for its regulation of body weight by regulating feeding behavior and energy expenditure. Additionally, there are demonstrated developmental effects of leptin on hypothalamic neurite outgrowth during postnatal juvenile life. In rodents, during the second week of postnatal life, serum leptin levels transiently increase several fold for several days and then return to normal low levels by postnatal day 16 (23). This juvenile “leptin surge” has functional significance in the hypothalamus, regulating neurite outgrowth and projections from the arcuate nucleus to the DMN, where RFRP-3 neurons reside (24, 25). In regards to RFRP-3, we found that a small subset of *Rfrp* neurons express the long form leptin receptor mRNA, suggesting that leptin could potentially act directly in those neurons. Supporting this possibility, we detected impaired *Rfrp* expression in adult *Obese* (Ob) mice (20), which produce a non-functional leptin peptide, rendering them morbidly obese in adulthood and reproductively incompetent (26, 27). Because there was a significant alteration of *Rfrp* mRNA expression in adult Ob mice, we tested whether *Rfrp* neuron maturation during juvenile life was dependent on proper leptin actions via the leptin surge. First, we measured serum leptin during postnatal development in normal female mice to determine if changes in leptin levels correlate with changes in *Rfrp* expression. We found that both leptin and *Rfrp* expression are similarly low at birth and the late neonatal period, and that both measures then start to rise in juvenile life. Serum leptin levels were highest just before and during the peak of *Rfrp* expression observed around postnatal day 12 (20), showing nice symmetry in the timing of developmental changes in these two measures. This experiment was followed up with an examination of *Rfrp* expression in postnatal Ob mice and their WT littermates. We hypothesized that leptin signaling is required, directly or indirectly, for the normal pattern of *Rfrp* neuron development in juveniles. However, despite the strong correlation between developmental *Rfrp* and leptin changes observed in normal mice, we found no differences between Ob and WT mice in any measure of *Rfrp* expression during postnatal development (20). Since leptin is not required for the developmental changes in *Rfrp* expression, other metabolic hormones, such as ghrelin or insulin (28–30), or reproductive hormones from maturing gonads (31–34), may be involved in driving the *Rfrp* developmental pattern during these postnatal ages.

## What Role Does RFRP-3 have in Peripubertal Life?

Given the dramatic developmental changes in *Rfrp* expression and neuronal activation during prepubertal and peripubertal life, it is possible that RFRP-3 regulates pubertal timing. Several investigators have used different methods to block RFRP-3’s production or action to test this hypothesis. First, a small interfering RNA was designed to knockdown RFRP-3 in prepubertal male rats (35). After 2 weeks of treatment, the knockdown successfully decreased RFRP-3 immunoreactivity and increased serum LH. However, this treatment was surprisingly unable to alter the timing of pubertal development (35). Second, a Gpr147 KO mouse line was developed in order to test the necessity of RFRP-3 signaling for mouse reproductive function (36). Gpr147 is the primary G-protein coupled receptor for RFRP-3, as determined by receptor–ligand binding and functional assays (7, 37). Gpr147 KO mice were confirmed to be unresponsive to exogenous RFRP-3, showing no decrease in LH after central RFRP-3 injections. Yet, surprisingly, Gpr174 KO females had no significant advancement or delay in pubertal timing, as measured by vaginal opening. Collectively, these two independent findings suggest that endogenous RFRP-3, acting on Gpr147, does not have a major role in timing puberty onset, at least in rodents. However, estrous cyclicity was slightly impaired in adult Gpr147 KO mice, which had a small but significant decrease in the time spent in diestrous, suggesting RFRP-3 may still have meaningful roles in reproduction in adulthood.

Despite the findings above, it remains possible that RFRP-3 may still have a developmental role in controlling LH secretion in juvenile or prepubertal mice, but not robustly altering puberty timing. Indeed, the same study that demonstrated normal puberty in Gpr147 KOs reported that prepubertal Gpr147 KO males have higher LH levels than their WT counterparts, suggesting that endogenous RFRP-3 is important in modulating prepubertal LH secretion. Interestingly, this genotype difference was normalized after puberty, with both KOs and WTs having similar adult LH levels (36). Therefore, RFRP-3 may not have a critical role in the timing of puberty onset or duration, but may still act to suppress LH secretion prior to the pubertal period, which would match the developmental stage when neural *Rfrp* levels are highest. Most recently, our lab examined the neuronal activation of multiple reproductive neuropeptides during the prepubertal and peripebuteral periods in C57BL6 female mice (21). Of the three reproductive neuropeptide systems examined, kisspeptin, neurokinin B and RFRP-3, *Rfrp* neurons were the only population to demonstrate a significant change in neuronal activation during the peripubertal period. More specifically, *c-Fos* co-expression, which marks recently activated neurons, was significantly higher in *Rfrp* neurons on PND 15 than on PND 21, reflecting lower neuronal activation at the latter age. Afterward, throughout subsequent pubertal ages (~PND 22-30), *c-Fos* induction in *Rfrp* neurons remained consistent around 40% versus the higher levels of neuronal activation ~60% at the earlier prepubertal age (PND 15). From these data, we infer that RFRP-3 secretion and actions on the reproductive axis are decreased at PND 21 and throughout puberty relative to a stronger inhibitory tone that potentially exists on PND 15. Additionally, we speculate that at some point between PND 15 and PND 21, there are either changes in the upstream stimulatory input onto RFRP-3 neurons or that the RFRP-3 neurons are intrinsically changing their activity and secretion by an undetermined mechanism.

Collectively, the above experiments suggest that RFRP-3 is unlikely to be a major player in substantially impacting the timing of pubertal onset or progression because (1) knocking down RFRP-3 production during puberty has no effect on pubertal time, (2) Gpr147 KO mice have normal timing of vaginal opening, and (3) any notable decrease in *Rfrp* gene expression or neuronal activation occurs multiple days, if not longer, before external markers of puberty are evidenced. Nonetheless, RFRP-3 may still prove to have role in restraining the reproductive axis before puberty can start or proceed. A recently published report demonstrated that RFRP-3 is able to suppress LH secretion in prepubertal female mice, but only in the presence of estradiol (22). It currently remains unclear why RFRP-3 had no stimulatory effect in the absence of estrogen. Regardless, this is the first report to examine the effect of RFRP-3 on LH secretion in prepubertal mice, and confirms the assumption that RFRP-3 can have bioactivity at these younger non-adult ages. Within the context of dramatic developmental changes in *Rfrp* expression and RFRP-3 neuronal firing discussed above, these new data suggest that RFRP-3 may have a role in maintaining the reproductive axis in a prepubertal quiescent state until other pubertal regulators initiate puberty.

## Conclusion

**Figure 2:**
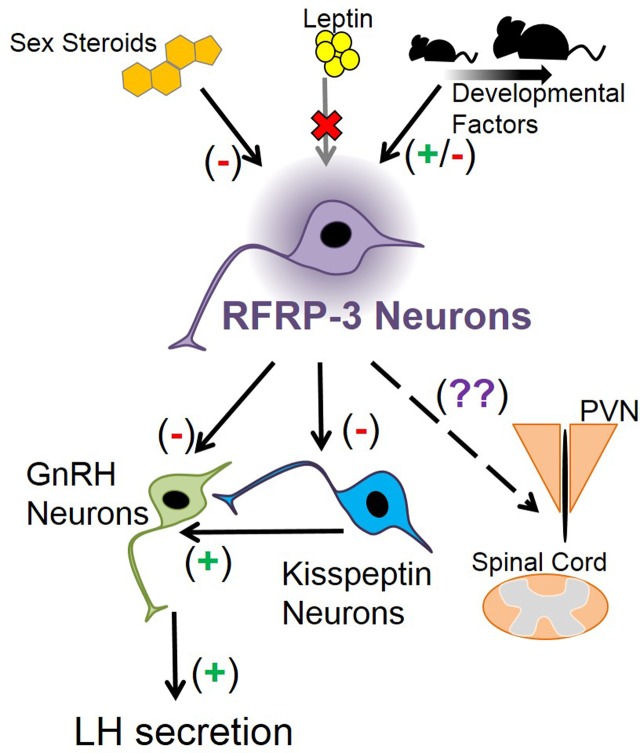
**Schematic depicting how *Rfrp* neurons may fit into mouse neuroanatomy and physiology**. Sex steroids are known to inhibit *Rfrp* expression, primarily through estrogen pathways, while leptin appears to have no major direct effect on *Rfrp* neurons, at least in development. There appear to be other developmental factors that regulate the development of *Rfrp* expression which are both stimulatory and inhibitory that remain to be determined. RFRP-3 neurons have efferents that may regulate LH secretion by acting directly on GnRH neurons, or indirectly on GnRH neurons through arcuate kisspeptin neurons or other yet-to-be identified neuronal populations. Other RFRP-3 neuronal target areas include the paraventricular nucleus (PVN) and the spinal cord, and RFRP-3 has unknown effects on these non-reproductive regions.

RFamide-related peptide-3 suppresses LH secretion when given exogenously. However, additional exploration into RFRP-3’s functional position in the mammalian reproductive axis has been limited. RFRP-3 neurons have distinct and quantifiable changes throughout development, hinting at a role in regulating puberty, but this was not corroborated by knockout and knockdown studies. Thus, RFRP-3’s developmental function is not immediately clear (Figure 2). Knockout mouse studies have shown that RFRP-3 neuron development is not strongly regulated by BAX-mediated apoptosis or by leptin, opening the possibility of other novel mechanisms that may influence the maturation of RFRP-3 neurons (Figure 2). Thus, RFRP-3’s specific developmental role is still being elucidated, leaving multiple avenues to explore. How does RFRP-3 regulate LH secretion in prepubertal animals, and why is it influenced by estrogen? Why does RFRP-3 neuron activation decrease after weaning? Why do some *Rfrp* neurons express high levels of *Rfrp* mRNA while others do not? Is there a functional significance to the robust *Rfrp* changes between birth and juvenile life and then again before adulthood? Answering these questions will further identify the role of RFRP-3 in development and reproduction in general.

## Conflict of Interest Statement

The authors declare that the research was conducted in the absence of any commercial or financial relationships that could be construed as a potential conflict of interest.
